# Influence of platinum group metal-free catalyst synthesis on microbial fuel cell performance

**DOI:** 10.1016/j.jpowsour.2017.11.039

**Published:** 2018-01-31

**Authors:** Carlo Santoro, Santiago Rojas-Carbonell, Roxanne Awais, Rohan Gokhale, Mounika Kodali, Alexey Serov, Kateryna Artyushkova, Plamen Atanassov

**Affiliations:** Department of Chemical and Biological Engineering, Center for Micro-Engineered Materials (CMEM), University of New Mexico, Albuquerque, NM 87131, USA

**Keywords:** Pyrolysis, Oxygen reduction reaction, Rotating ring disk, Microbial fuel cell, Reproducibility

## Abstract

Platinum group metal-free (PGM-free) ORR catalysts from the Fe-N-C family were synthesized using sacrificial support method (SSM) technique. Six experimental steps were used during the synthesis: 1) mixing the precursor, the metal salt, and the silica template; 2) first pyrolysis in hydrogen rich atmosphere; 3) ball milling; 4) etching the silica template using harsh acids environment; 5) the second pyrolysis in ammonia rich atmosphere; 6) final ball milling. Three independent batches were fabricated following the same procedure. The effect of each synthetic parameters on the surface chemistry and the electrocatalytic performance in neutral media was studied. Rotating ring disk electrode (RRDE) experiment showed an increase in half wave potential and limiting current after the pyrolysis steps. The additional improvement was observed after etching and performing the second pyrolysis. A similar trend was seen in microbial fuel cells (MFCs), in which the power output increased from 167 ± 2 μW cm^−2^ to 214 ± 5 μW cm^−2^. X-ray Photoelectron Spectroscopy (XPS) was used to evaluate surface chemistry of catalysts obtained after each synthetic step. The changes in chemical composition were directly correlated with the improvements in performance. We report outstanding reproducibility in both composition and performance among the three different batches.

## Highlights

• Iron-Nicarbazin was synthesized using Sacrificial Support Method.
• Temperature treatment and acid treatment modify the surface chemistry.
• Etching and second pyrolysis affect positively the performances.
• Maximum MFC power output achieved in this work was 214 ± 5 μWcm^−2^.
• High reproducibility was detected within three independent batches.

## 1 Introduction

Bioelectrochemical systems are fascinating technologies in which electroactive microorganisms consume a variety of organic compounds and release electrons directly on the anode electrode [1], [2]. Microbial fuel cell (MFC) is by far the most studied with the perspective of generating electricity for practical applications and removing organics and pollutants from the electrolyte [3], [4]. One of the biggest problems related to the electrochemical performances of bioelectrochemical systems (BESs) is certainly the sluggish cathodic reaction. Several oxidants were utilized and studied, but by far oxygen represents the best option due to several intrinsic characteristics such as high reduction potential and the natural availability without a cost associated with it [5], [6], [7]. Oxygen reduction reaction (ORR) suffers from numerous severe limitations when it occurs in neutral media, and therefore an optimization of the catalyst is needed to accelerate the process [8], [9], [10]. First, high activation overpotentials exist, being as high as 50–100 mV when enzymes are utilized [11], [12], [13], [14], 200–300 mV in the case of platinum based group metal (PGM) catalysts [15], [16] or platinum group metal-free (PGM-free) catalysts [17], [18], [19], [20], [21], [22], and even larger in the case of bacterial catalyst [23], [24], [25] or carbonaceous materials [26], [27], [28], [29], [30], [31], [32], [33]. Second, the ORR reaction kinetics is very slow mainly due to the neutral pH, in which both H^+^ and OH^−^ are present in low concentration and both of them participate directly as reactants of ORR, the first one via the acidic pathway and the second one via the alkaline pathway [5], [6], [25]. Moreover, unfortunately, due to the presence of biotic matter on the anode electrode, the increase in temperature, which can usually be used to enhance the kinetic rate of ORR, cannot be utilized to a full extent as it can degrade the entire microbiological system [34], [35]. To be competitive with other energy sources or wastewater treatment systems, the cost of the catalyst in a low current/energy producing system must be considered. Both enzymes and platinum-based electrocatalysts are expensive, and moreover, are not durable at conditions in which pollutants and anions are present in abundance [36], [37], [38], [39], [40]. Bacterial catalysts also can not be used due to their low kinetics and high activation overpotentials [23], [24], [25], making carbonaceous based and PGM-free catalysts the only suitable candidates to be utilized in catalyzing ORR in MFCs. The application of both types of catalysts is increasing over time as was summarized in a recent review [16], [41], [42], [43]. Considerable effort was devoted to using commercial high surface area carbonaceous materials such as activated carbon (AC) [44], [45], [46], [47], [48], [49], [50], modifying it to increase the surface area or to functionalize it to enhance ORR [44], [45], [46], [47], [48], [49], [50]. Moreover, the ongoing research is focused on developing, fabricating and studying new carbonaceous materials such as graphene [23], [30], [51], [52], carbon nanotubes and carbon nanofibers [53], [54] etc. All of these materials have outstanding properties such as high surface area, high resistance to corrosion, high mechanical strength, relatively high electrical conductivity in common. Carbonaceous materials demonstrated high durability in long terms operations with losses identified in 15–30% over one-year operations [49], [55].

In parallel, application of PGM-free materials has risen significantly mainly due to the relatively affordable cost of the high performing catalysts that are made out of transitional metals such as Fe, Mn, Ni and Co. Three types of PGM-free materials were recently used in MFCs [16], [42], [43]. The first type is based on the utilization of transitional metal oxides such as Fe, Co, Ni, etc [56], [57], [58], [59], [60], [61]. The improvement compared to AC is certainly important, but still, there are obvious limitations in performance [56], [57], [58], [59], [60], [61]. The second type is based on the use of non-pyrolyzed macrocyclic organic compounds such as porphyrins, phthalocyanine, etc. with the incorporation of the metal center such as Fe, Co, Ni [62], [63], [64], [65], [66], [67], [68], [69], [70]. Despite the high performances achieved, the main limitation is the high cost of the macrocyclic organic compounds that hinder their introduction in the commercialization world [62], [63], [64], [65], [66], [67], [68], [69], [70]. The third type is based on the high-temperature synthetic method in which metal salts and an organic rich in nitrogen precursors are pyrolyzed at a temperature above 900 °C [71], [72], [73], [74], [75], [76], [77]. The last type is the most adopted for fabricating catalysts utilized in MFCs [26], [42], [43]. Fe-based catalysts seem to be the most promising since they perform better than Co-based catalysts [17], [18]. Mn-based [17], [18] and Ni-based [17], [18] had high performances compared with bare AC but their performances were lower compared to Fe- and Co-based catalysts.

The method we adopted to produce our catalyst is based on a technique named Sacrificial Support Method (SSM) [79], [80]. This method was used to create catalysts that were previously tested in MFCs [37], [38], [47], [71], [74], [75], [76], [78]. SSM technique consists of mixing metal salt and organics precursors with monodispersed silica acting as a template. The etching of the silica using aggressive acidic conditions allows creating a three-dimensional structure.

In this work, a catalyst was prepared using SSM technique following six steps. The organic precursor (Nicarbazin, N-C source) was mixed with the metal salt (iron nitrate, metal source) and the templating silica particles (step 1). The mixture was then pyrolyzed in reducing atmosphere (step 2). After pyrolysis, the mixture was ball-milled (step 3) and then silica was etched (step 4). The second pyrolysis was applied to the sample (step 5) and then the obtained material was further ball-milled (step 6). Three separate batches were used to fabricate the catalysts. A small quantity of material was saved after every synthesis step. The electrocatalytic activity toward ORR of the catalyst produced after each step in three different batches was evaluated using rotating ring disk electrode (RRDE) in neural media. Those measurements allowed measuring the disk and ring current and identifying the H_2_O_2_ produced as well as the electron transfer mechanism involved. The catalysts were then incorporated into air-breathing cathodes and tested in working microbial fuel cells. After every synthetic step, the surface chemistry of the catalyst was analyzed by X-ray Photoelectron Spectroscopy (XPS) and it was then related to the electrochemical performance of the catalyst both in RRDE and in MFCs.

## 2 Materials and method

### 2.1 Catalyst preparation

Three different batches of the PGM-free catalyst were prepared, and particularly, a sample of the product was collected and saved for testing for each step of the synthesis.

The synthesis consists of six main steps identified as 1) mixing; 2) the first pyrolysis; 3) ball milling; 4) etching; 5) the second pyrolysis; 6) ball milling. The mixing step consists in combining 55.6% (wt./wt.) of the organic precursor (Nicarbazin, Sigma-Aldrich, 98%), 11.0% (wt./wt.) of in-house prepared Stöber spheres, 13.9% of LM-150 fumed silica (Cabot); 13.9% of OX-50 hydrophilic fumed silica (Aerosil) and 5.6% of iron nitrate nonahydrate (Sigma-Aldrich, 99.95%). The mixing was initialized adding deionized water. The obtained mixture was stirred overnight at a constant controlled temperature of 45 °C and 300 RPMs. After becoming a dry solid mixture, a further dry treatment was done using an oven at 85 °C for additional 16 h. The dry mixture was then ball milled with agate glassware at 350RPM for 30 min, and a sample of each batch was collected (labeled A1, A2 and A3 respectively) (Table 1). These three samples were selected for the surface chemistry analysis only, but not for the electrochemical measurements. This decision was dictated by the fact that the mixture was not electrically conductive (organic, inorganic precursors and a substantial amount of non-conductive SiO_2_).

**Table 1:** Description of the samples studied and the synthesis steps done.

Sample number	1st pyrolysis	Ball milling	etching	2nd pyrolysis	Ball milling	Sample abbreviation
1,2,3						A
4,5,6	x					1P
7,8,9	x	x				1PB
10,11,12	x	x	x			1PBE
13,14,15	x	x	x	x		1PBE2P
16,17,18	x	x	x	x	x	1PBE2PB

After the fine powdered mixture was obtained from the previous step, it was subjected to the first heat treatment (HT). The powder was placed in a porcelain boat and introduced into a quartz tubular furnace. The tubular configuration allowed a reductive atmosphere of 7 at% hydrogen balanced with ultra high purity (UHP) nitrogen flow (100 cm^3^ min^−1^). The furnace was preheated at 525 °C, and then the quartz tube containing the sample was placed in the hot zone of the furnace. Then, the temperature was increased to 900 °C by a ramp rate of 75 °C min^−1^. Once reached the designated point, the temperature was set at 975 °C with a slower ramp rate of 10 °C min^−1^. The sample was left at this final temperature for 45 min, after which the quartz tube was removed from the furnace and let to cool down to ambient temperature while maintaining the reductive atmosphere flow. The collected samples after this first heat treatment are 1P-1, 1P-2 and 1P-3 (Table 1).

The heat-treated samples were then ball milled in agate jar at 350 RPM for 30 min, and the ball-milled samples for each batch were separately collected. The samples are named 1PB-1, 1PB-2 and 1PB-3 accordingly to Table 1. The obtained powders were etched for three days in a 2:1 mixture of hydrofluoric acid (HF, Solvay, 25 wt%) and nitric acid (HNO_3_, Sigma-Aldrich, 35 wt%), with the scope of removing the silica templating and the metal-derived particles, formed during the reductive heat treatment. After the etching step, the samples were thoroughly washed with DI water till achieving neutral pH. The obtained samples were then dried for 16 h at 85 °C to remove any water content. These etched powders were named as 1PBE-1, 1PBE-2 and 1PBE-3.

The samples were also subject to a second heat treatment that carried out similarly than the one presented earlier with the only difference that the gas was 10 at% ammonia balanced with nitrogen (flow rate 100 cm^3^ min^−1^). The samples after the second heat treatment were labeled as 1PBE2P-1, 1PBE2P-2 and 1PBE2P-3 (Table 1).

At last, a final ball milling was carried out for the samples in agate glassware at 50 Hz for 30 min, and the collected samples were labeled 1PBE2PB-1, 1PBE2PB-2, 1PBE2PB-3 (Table 1). The catalysts analyzed in this work are shown in Table 1.

### 2.2 Surface chemistry analysis

The surface chemistry of the catalyst was determined using x-ray photoelectron spectroscopy (XPS). Kratos Ultra DLD spectrometer was used. Spectra were obtained from three areas using monochromatic Al Kα source at 225 mW. Survey and C 1s, O 1s, N 1s and Fe 2p high-resolution spectra were obtained at 80 and 20 eV pass energy, respectively. For all samples, no charge neutralization was used. Data analysis and quantification were performed using CasaXPS software. A 70% Gaussian/30% Lorentzian line shape was utilized in the curve-fit of spectra. The linear background was used for quantifying atomic composition except for Fe 2p spectra, for which Sherley background was used. All spectra were fitted using previously adapted set of peaks [[69], [75], [81], [82]].

### 2.3 Rotating ring disk electrode (RRDE) measurements

Rotating ring disk electrode (RRDE) technique was used to determine the kinetics parameters of the catalysts investigated. The ink for each catalyst was prepared by mixing 5 mg of the catalyst with 1 mL of solution consisted of 8.5 part of the liquid solution was composed of a mixture of isopropanol and distilled water in ratio 1:4 respectively and the remaining 1.5 part of the solution was composed of 0.5 wt% of Nafion solution. The ink was sonicated for 5 min and then shaken for 3 min. This latter procedure was repeated 3 times. The ink was deposited onto the disk using a micropipette and left air-dried in natural environment till fully dried. The catalyst loading on the disk was 0.175 mgcm^−2^. The same ink was prepared for AC used as control and drop casted on the disk electrode with the equal loading used for the catalysts. The experiments were done using a circumneutral electrolyte (pH 7.5) composed by 0.1 M of potassium phosphate with the addition of 0.1 M KCl. The electrolyte was initially saturated with pure oxygen that was flushed using an air diffuser into the liquid for at least 20 min before running the electrochemical tests. Linear sweep voltammetry (LSV) was used for characterizing the catalysts. The operations were carried out scanning between 1.0 (vs. RHE) and 0.0 (vs. RHE) using a scan rate of 5 mVs^−1^. The configuration was a three electrodes configuration with the disk electrode (area of 0.2475 cm^2^) with the drop-casted catalyst being the working electrode, a graphite rod as a counter electrode and Ag/AgCl electrode (3 M KCl) as the reference electrode. Onset potentials, half wave potentials, and limiting currents were identified within the three different catalyst batches during the five steps of synthesis. Moreover, disk (I_disk_) and ring (I_ring_) current was measured to determine hydrogen peroxide yield and the number of electrons transferred according to equation (1) (eq. (1)) and equation (2) (eq. (2)) respectively.

(1) $$\%H_{2}O_{2}=\;\frac{200\;\times \;\frac{I_{ring}}{N}}{I_{disk}+\frac{I_{ring}}{N}}$$

(2) $$n=\frac{4I_{disk}}{I_{disk}+\frac{I_{ring}}{N}}$$

### 2.4 Cathode preparation

Once the catalyst was characterized, each catalyst was incorporated into an air-breathing cathode and tested in operating MFC. The air-breathing cathode was fabricated by pressing a mixture on a stainless steel mesh (McMaster, USA) working as a current collector. As shown before [74], [75], [76], [78], a mix of activated carbon (AC), carbon black (CB) and a solution of polytetrafluorethylene (PTFE) was inserted into a ball-mill and ball-milled for at least 5 min. The solid mixture had a composition in weight percentage that was 70%, 10% and 20% respectively. A quantity of 400 mg of the above mixture was homogenized with 20 mg of each catalyst. The quantities loadings (15 mg) were weighed using a precise balance. The obtained black powder was then inserted into a metallic pellet dye and then pressed at 2 mT for 5 min. Each cathode contains a loading of AC/CB/PTFE that was 40 mg cm^−2^ and a Fe-N-C catalyst loading that was 1.5 mg cm^−2^.

### 2.5 Microbial fuel cell (MFC) operations and electrochemical performances

The cathode was screwed on a modified Pyrex glass bottle that was customized with a lateral hole. The MFC was then filled with 125 mL of electrolyte composed of 50% in volume of activated sludge (Albuquerque Southeast Water Reclamation Facility, New Mexico, USA) and the remaining 50% in volume with 0.1 M potassium phosphate. Sodium Acetate (NaOAc) in the concentration of 3 g L^-1^ was added into the chamber as bacterial feedstock. Two cylindrical carbon brushes (Millirose, USA) of diameter 3 cm and height of 3 cm were used as an anode electrode. The anodes were already well working with already established electroactive biofilm and were moved from operating MFCs into the new MFCs adopted for this experiments. Same anodes were used during the experiments. The surface area of the cathode exposed to the solution was 2.9 cm^2^. The catalyst layer was exposed to the liquid solution. The current collector was exposed to the natural atmosphere. After the anodes were transferred into the new system, the MFC was left in open circuit voltage (OCV) to allow the establishment of anaerobic conditions. After at least 2 h, when the OCV was stable, polarization curves were performed on the system. Two potentiostats were used during this experiment. One potentiostat (Biologic SP50) was designated to run the linear sweep voltammetry (LSV) in two-electrodes configuration with the working channel connected to the cathode and the counter channel (short circuited with the reference channel) connected to the anode. LSV was run from OCV to 0 mV at a scan rate of 0.2 mVs^−1^. The second potentiostat was used to read the potential of each electrode versus an Ag/AgCl 3 M KCl connecting the working channel to the cathode, the counter channel to the anode and the reference channel to the reference electrode. One cathode for each of the different three batches and the five different pyrolysis steps was tested in MFC.

### 2.6 Statistical analysis and interpretation

The electrochemical data of interests were: i) onset potential; half wave potential and limiting current from the data measured through RRDE; ii) OCV, short circuit current density, maximum power density and cathode potential at a current density of 600 μWcm^−2^ were used from the data collected through the MFCs data. Hydrogen peroxide produced during the RRDE experiments (eq. (1)) and the electron transferred according with eq. (2) was also taken into account. Those electrochemical data were correlated with the surface chemistry data obtained through XPS analysis. Principal component analysis (PCA) (in PLSToolbox 8.2 in Matlab) auto scaling was used to visualize correlations between variables and samples. PCA is a multivariate method of converting a large number of variables into new mathematical variables called principal components. The first principal component (PC 1) captures the greatest possible variance in the data and the second (PC 2), orthogonal to the first, captures the second largest variance. A biplot displays both, scores for each sample and the loadings for each variable, visualizing clustering of samples based on similarities. This statistical approach enables the identification of the most or least important surface chemistry descriptors for a given performance output.

## 3 Results and discussion

### 3.1 Catalyst surface chemistry

**Fig. 1:**
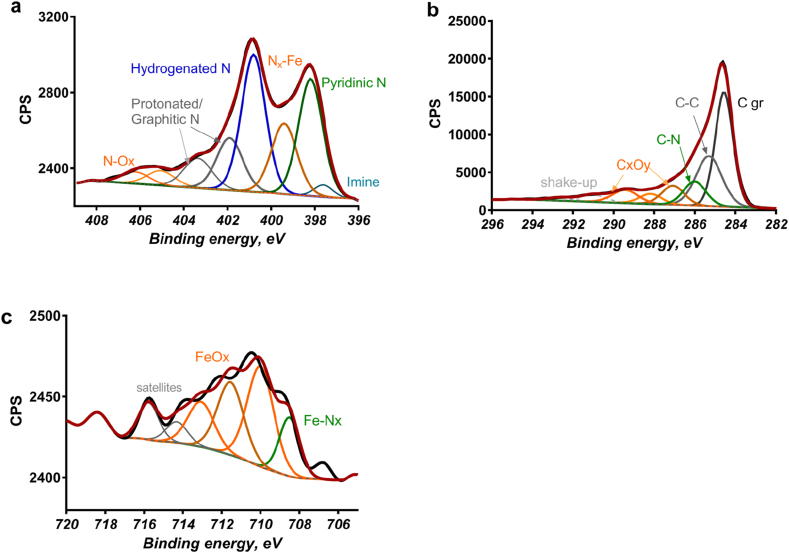
XPS high-resolution N 1s (a), C 1s (b) and Fe 2p (c) spectra for the sample after the first pyrolysis, ball milling, etching and second pyrolysis (1PBE2P-1). The sample was selected from the first of the three batches.

Fig. 1 shows high-resolution N 1s (Fig. 1a), C 1s (Fig. 1b) and Fe 2p (Fig. 1c) spectra for sample 1PBE2P-1. The average elemental composition (Table 2) and relative distribution of nitrogen (Table 3), carbon (Table 4), and iron (Table 5) chemical species of the three batches after each synthesis step are presented. The data were extracted from fitted spectra for all samples. The data related on the surface chemistry of each separate sample are presented in details in Tables S1, S2, S3 and S4.

**Table 2:** Average elemental composition using XPS.

	C %	N %	O %	Fe %
1P	62.8 ± 4.6	2.7 ± 0.5	34.4 ± 4.3	0.20 ± 0.04
1PB	68.6 ± 0.9	2.6 ± 0.2	28.7 ± 0.8	0.21 ± 0.05
1PBE	85.2 ± 0.6	4.4 ± 0.3	10.3 ± 0.9	0.09 ± 0.01
1PBE2P	92.0 ± 0.2	4.3 ± 0.2	3.6 ± 0.2	0.15 ± 0.02
1PBE2PB	90.3 ± 0.9	3.8 ± 0.4	5.93 ± 1.3	0.13 ± 0.01

**Table 3:** Average relative distribution of nitrogen using XPS.

	N imine	N pyridinic	N_x_-Fe + amines	N-H	Ngr-N^+^	NO_x_
1P	2.7 ± 0.3	22.3 ± 1.8	18.2 ± 0.6	28.6 ± 0.9	21.5 ± 1.1	6.6 ± 1.0
1PB	2.1 ± 0.6	19.4 ± 1.7	20.3 ± 0.9	29.8 ± 1.0	23.0 ± 1.6	5.3 ± 0.5
1PBE	0.8 ± 0.7	17.7 ± 0.7	18.5 ± 0.8	29.3 ± 0.1	15.7 ± 0.7	17.9 ± 0.3
1PBE2P	2.7 ± 0.6	25.3 ± 0.5	14.9 ± 0.8	29.8 ± 1.0	19.9 ± 1.5	7.4 ± 0.6
1PBE2PB	2.4 ± 0.1	25.8 ± 0.2	16.2 ± 0.8	29.8 ± 1.4	19.0 ± 0.9	6.8 ± 0.6

**Table 4:** Average relative distribution of carbon using XPS.

	C gr	C-C	C-N/C-O		C=O	COOH
1P	30.4 ± 6.3	13.4 ± 2.3	29.4 ± 4.4	7.5 ± 0.5	7.1 ± 1.5	8.0 ± 0.7
1PB	27.6 ± 0.6	21.6 ± 4.2	23.9 ± 2.7	7.6 ± 0.3	6.8 ± 1.0	8.6 ± 0.4
1PBE	39.9 ± 0.2	19.1 ± 0.2	14.4 ± 0.5	8.0 ± 0.4	6.5 ± 0.4	6.2 ± 0.4
1PBE2P	40.6 ± 0.9	18.6 ± 1.9	13.5 ± 0.6	8.5 ± 0.8	5.6 ± 0.4	6.2 ± 0.1
1PBE2PB	37.5 ± 1.8	23.8 ± 1.4	12.9 ± 1.2	8.6 ± 0.2	4.7 ± 0.4	6.8 ± 0.5

**Table 5:** Average relative distribution of iron using XPS.

	Fe-N_x_	FeO_x_
1P	12.2 ± 1.6	87.8 ± 1.6
1PB	10.9 ± 2.2	89.1 ± 2.2
1PBE	19.4 ± 5.7	80.6 ± 5.7
1PBE2P	23.0 ± 3.2	77.0 ± 3.2
1PBE2PB	21.6 ± 4.4	78.4 ± 4.4

The composition of samples after the 1st pyrolysis and subsequent ball milling is very similar. This is quite expected because this procedure of does not affect the surface chemistry of the samples. The high amount of oxygen is present, which is also manifested by a significant peak in C 1s spectra due to C-O species at 286. eV. A large amount of graphitic nitrogen is also present in comparison with other samples. Iron oxides are present in excessive amounts in these samples as well (Table 5).

Interestingly, etching the samples causes major changes in the surface chemistry (Table 2, Table 3, Table 4, Table 5). Three times smaller amounts of oxygen are present after leaching (Table 2) indicating the removal of the iron oxides and that cleaning the surface of carbon oxides is an essential part of leaching. This causes more carbon detected and higher surface concentrations of nitrogen. Higher amounts of graphitic and aliphatic carbon are also observed. Iron is being leached out as well, during this step as twice smaller amount of Fe is detected. At the same time, nitric oxides are increased for etched samples, due to the use of nitric acid in leaching (Table 3).

The composition of samples after the second pyrolysis and ball milling is very similar. The second pyrolysis serves as another cleaning step, as the even higher amount of carbon and a smaller amount of oxygen is detected in these samples (Table 2). The second pyrolysis removes surface oxides, even more, allowing more of iron coordinated to nitrogen exposure to the surface. This is confirmed by an increase in Fe-N_x_ peak in Fe 2p spectra (Fig. 1). The nitric oxides are also being removed during the second pyrolysis. This confirms that nitric oxides were due to a not complete washing after the leaching step in which nitric acid was used. Higher amounts of pyridinic nitrogen are observed (Table 3). Smaller amounts of nitrogen centers coordinated to iron detected in samples after the second pyrolysis from N 1s spectra (which is contradictory to observations from Fe 2p spectra) are because amines are contributing to the same binding energy and they are being removed during second pyrolysis (Table 3).

### 3.2 RRDE analysis

As mentioned in the Introduction Section, ORR can follow two pathways as function of the electrolyte pH in which the experiments are performed. The acidic pathway can follow a 2e^−^ transfer mechanism producing H_2_O_2_ or a direct 4e^−^ transfer mechanism with H_2_O as final product or a combined 2x2e^−^ transfer mechanism with the reaction intermediate chemically or electrochemically transformed to H_2_O. Instead the alkaline pathway can follow a 2e^−^ transfer mechanism producing HO_2_^−^ or a direct 4e^−^ transfer mechanism with final product OH^−^. Also in this specific case, a combined 2x2e^−^ transfer mechanism with the reaction intermediate chemically or electrochemically transformed to OH^−^. Rotating ring disk electrode (RRDE) is a technique used to study the kinetic towards ORR of a catalyst in a media with defined pH conditions. In parallel to the disk current, also the ring current can be measured and related with the intermediate obtained during the oxygen reduction reaction (ORR). The presence of peroxide can be an indicator of the fact that ORR does not follow a direct 4e^−^ transfer mechanism but it can follow a 2e^−^ or 2x2e^−^ transfer mechanism. Generally, a 4e^−^ transfer mechanism is preferred and it is more efficient compared to a 2e^−^ or a 2x2e^−^ transfer mechanism. In fact, if the reaction follows a 2e-transfer mechanism, more reactant (oxygen) is necessary to complete the red-ox reactions and therefore is less effective than the direct 4e^−^. In operating MFCs, intermediates can be deleterious for the electroactive bacteria and therefore unwanted and undesired reaction products.

**Fig. 2:**
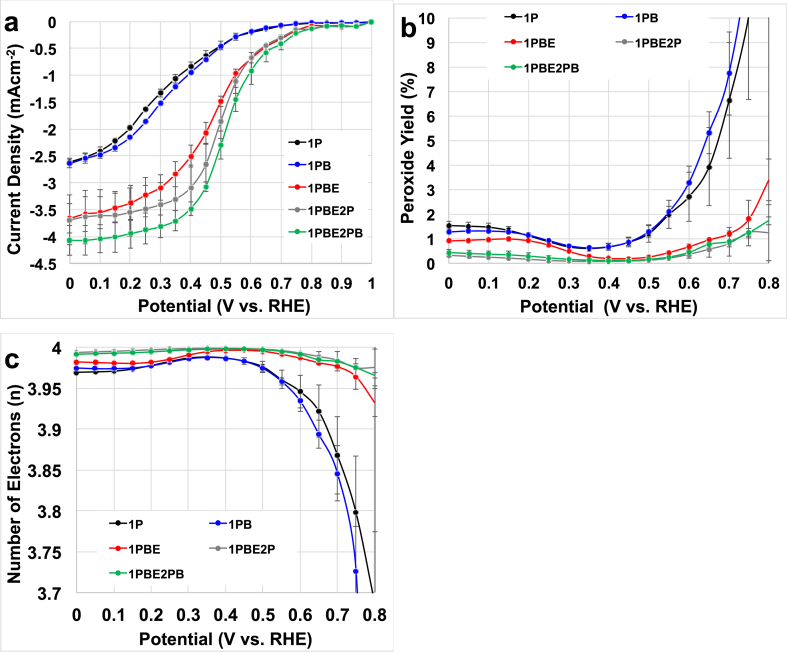
Average disk current (a), average peroxide yield (b) and average number of electrons transferred (c) for the catalysts synthesized for each step.

Average disk current (Fig. 2a), peroxide production (Fig. 2b) and electron transfer mechanism (Fig. 2c) among the three batches are presented and discussed here. Clearly, different synthesis steps affect the performances (Fig. 2a). In fact, the onset potential remains stable varying between 0.79 and 0.85 V (vs RHE) with average value of 0.82 ± 0.02 V (vs RHE), but the half wave potential and the limiting current increased with the steps (Fig. 2a). AC had a much lower OCP that was measured in ≈0.5 V (vs RHE) (Fig. S1). The half wave potential increased from 0.28 ± 0.02 V (vs RHE) to 0.49 ± 0.02 V (vs RHE) within the five synthesis steps. Two main stages of improvement are observed: the first one after the etching and the second one after the second pyrolysis (Fig. 2a). Both steps of ball milling processes did not affect the performances in the significant positive way (Fig. 2a). The half wave potential after the first pyrolysis was 0.28 ± 0.02 V (vs RHE) and interestingly it was similar to AC that was 0.27 V (vs RHE) (Fig. S1). The half wave increased to 0.48 ± 0.01 V (vs RHE) (after etching) and further to 0.49 ± 0.02 (vs RHE) (after the second pyrolysis). Limiting current also increased from 2.5 ± 0.1 mAcm^−2^ to 4.05 ± 0.15 mA cm^−2^ after the five synthetics steps (Fig. 2a). AC had a slightly smaller limiting current measured at 2.2 mAcm^−2^ (Fig. S1). Generally speaking, the ball milling processes did not affect the performances output. It must be noticed that the peroxide produced by the iron-based catalysts was very low with measured values lower than 2% (Fig. 2b). A decrease in the production of the intermediate was determined (Fig. 2b). Interestingly, it can be observed that the peroxide produced decreases with the potential indicating that the catalyst is further reducing the peroxide (Fig. 2b). Moreover, the peroxide yield slightly decreased after synthetic steps stating that each step is reducing the amount of intermediate-producing sites and favor the generation of the peroxide-reducing sites (Fig. 2b). This underlines that each step during the catalyst preparation is necessary for decreasing peroxide production. Interestingly, the control AC produced high yield of H_2_O_2_ that decreased from 77% at 0.5 V (vs RHE) to 47% at 0 V (vs RHE) (Fig. S1).

In summary, AC follows a straight 2e^−^ transfer mechanism with high peroxide produced (Fig. S1). Considering the Fe-NCB, certain synthetic steps are more influential than others. Both ball milling steps were not as important as the etching and the second pyrolysis. In fact, the second pyrolysis decreased the peroxide yield in a more significant way (Fig. 2b). Same considerations can be done for the electron transfer mechanisms. As the peroxide yield decreased with the potential, a 2x2e-transfer mechanism can be speculated. The number approach more and more 4e-with each step in the pyrolysis process (Fig. 2c). Once again, the main gain is visible after the samples are etched and undergo the second pyrolysis (Fig. 2c).

Within the same preparation batch, performances (Fig. S2), peroxide production (Fig. S3) and electron transfer mechanism (Fig. S4) follow the same trend (1P < 1PB < 1PBE < 1PBE2P < 1PBE2PB) during the different synthesis steps. High reproducibility in terms of performances (Fig. S5), peroxide production (Fig. S6) and electron transfer mechanism (Fig. S7) among the three batches during a single pyrolysis step is here shown.

### 3.3 Performances in microbial fuel cells

Polarization curves, power curves, anode and cathode polarizations of each sample from each batch separated during every synthesis step are presented in the Supporting Information (Figs. S8, S9, S10, S11 and S12). The reproducibility among the three different batches was very high, and therefore the average polarization curves, power curves and anode and cathode polarization curves are discussed.

**Fig. 3:**
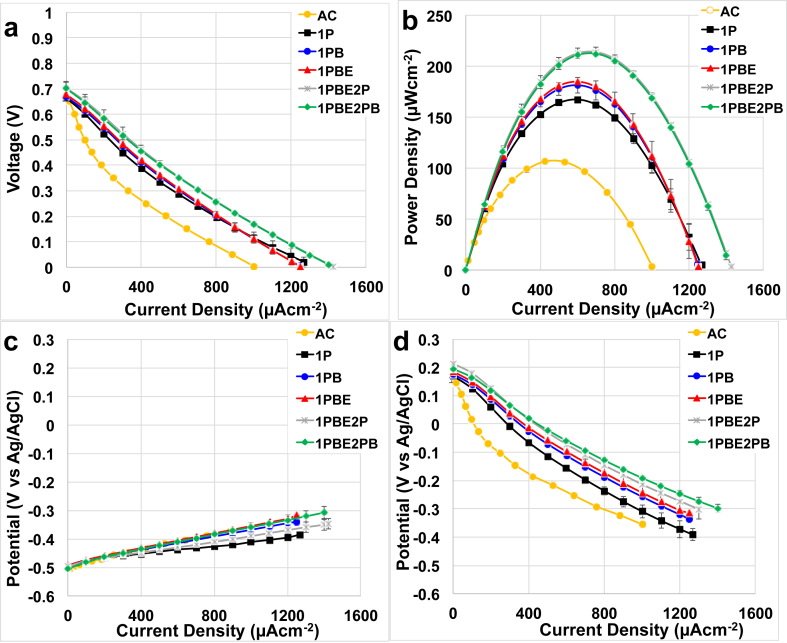
Polarization curves (a), power curves (b), anode (c) cathode (d) polarization curves of the catalysts integrated into air-breathing cathodes.

Average polarization curves for each the catalyst exposed to different synthesis treatment and then incorporated into air-breathing cathode MFCs are presented (Fig. 3a). The performances were compared with control cathodes only fabricated with AC, carbon black (CB) and PTFE as a binder. The latter is considered state of the art for MFC systems. From the polarization curves, it must be noticed that every Fe-based cathode MFCs have similar open circuit voltage (OCV) that was 683 ± 15 mV. In contrast to this, AC cathode MFCs had lower OCV quantified in 657 ± 5 mV. Short circuit current increased from 1030 μAcm^−2^ for AC cathode to 1266 μAcm^−2^ for Fe-based cathode after the first pyrolysis and reaching 1425 μAcm^−2^ for Fe-based cathode at the last stage of catalyst fabrication (Fig. 3a). It was noticed that the large increase in short circuit current was achieved after the second pyrolysis (Fig. 3a). The ball milling performed after the second pyrolysis did not increase the parameter considered (Fig. 3a). Using the power density obtained after the first pyrolysis as a baseline for comparison, power curves showed that the peak of power density increased slightly after ball-milling and it did not change after etching (Fig. 3b). In fact, the power density after the first pyrolysis was 167 ± 2 μWcm^−2^ that slightly increased after ball-milling (181 ± 8 μWcm^−2^) and remained stable after etching at 185 ± 3 μWcm^−2^ (Fig. 3b). The second pyrolysis gave a more detectable enhancement in performances with values measured of 214 ± 5 μWcm^−2^. The ball-milling after the pyrolysis did not provide a significant advantage, in fact, the performances remained stable at 212 ± 3 μWcm^−2^ (Fig. 3b). The advantage in terms of power percentage increase was 8% from the first pyrolysis to ball milling and after etching (10%). When the second pyrolysis was applied, the power density jumped up by 26%. The last ball milling did not give any significant catalytic activity enhancement. The overall advantage from the first pyrolysis to the last step (ball milling after the second pyrolysis) was quantified as 28%. Comparing to the power density achieved using AC cathode (106 ± 2 μWcm^−2^), the advantage observed when the Fe-NCB catalyst was added is quantified as 57%, 70%, 74%, 101% and 104% for each synthesis step respectively. This result underlines the need of utilizing low-cost PGM-free cathode catalyst to enhance the low performances of an MFC. Polarization curve of anode and cathode measured during the overall polarization curves showed very similar and comparable performances for the anode polarization, expected since the same anodes were used (Fig. 3c). An enhancement in cathodic activity with each synthesis step was visible during the cathode polarization curves (Fig. 3d) indicating that the catalytic activity of the cathode was the main reason for the enhancement of the overall MFC polarization curve (Fig. 3a). The cathode potential measured at a current of 600 μAcm^−2^ increased with the synthesis steps and it was −0.156 ± 0.014 V (vs Ag/AgCl) for 1P, −0.112 ± 0.009 V (vs Ag/AgCl) for 1PB, −0.098 ± 0.003 V (vs Ag/AgCl) for 1PBE, −0.072 ± 0.011 V (vs Ag/AgCl) for 1PBE2P and −0.059 ± 0.009 V (vs Ag/AgCl) for 1PBE2PB.

### 3.4 Structure-to-property analysis

**Fig. 4:**
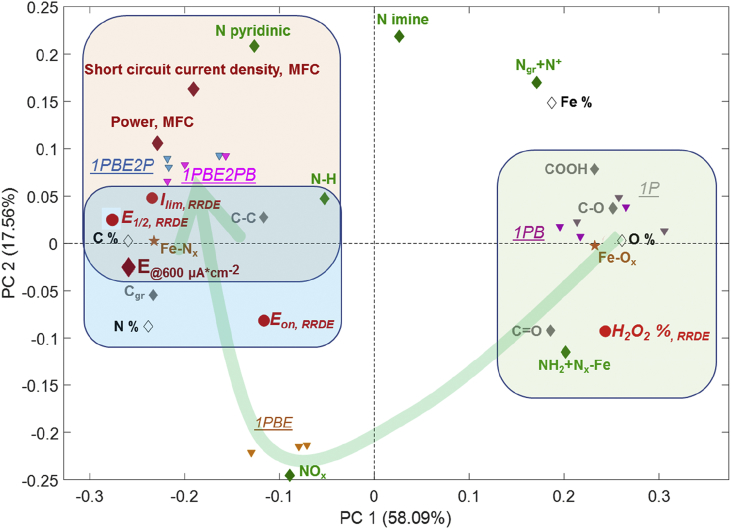
Principal Component Analysis (PCA) for the catalysts prepared during the different steps of pyrolysis. Surface chemistry, RRDE performances, and MFCs performances are considered.

Fig. 4 shows the PCA biplot identifying clustering between the samples based both on surface chemistry and resulting electrochemical performance. The starting chemistry begins with samples after the first pyrolysis and ball milling which have the highest amount of oxygen, surface oxide groups, iron oxides, and amines. These samples have the largest amount of hydrogen peroxide yield detected by RRDE. This is the critical observation that shows that in neutral media there is a bifunctional mechanism and surface oxides and iron oxides that are present in the catalyst will produce hydrogen peroxide.

As discussed above, the etching step caused the introduction of nitrous oxides. Interestingly, the chemistry evolves to create powder with a larger amount of carbon, nitrogen, nitrogen coordinated to the metal. The second pyrolysis is removing excess of surface oxides, iron oxides, amines and nitrous oxides, and better performance in both RRDE and MFC is observed. In RRDE performances, the contribution of iron centers coordinated to nitrogen and overall carbon with mostly graphitic character, manifesting higher conductivity, is translated into higher onset potential, half-wave potential and limiting current density. In operating MFC, however, the role of pyridinic nitrogen is also very significant. Pyridinic nitrogen is correlated with higher short circuit current densities, cathode potential at a current density of 600 μWcm^−2^ and power densities obtained in microbial fuel cell tests.

The significant parameters describing the surface chemistry of the catalyst during each step (Table 2, Table 3, Table 4) were identified using PCA (Fig. 4) and related to the electrochemical performances in RRDE (Fig. 2) and in MFC performances (Fig. 3).

**Fig. 5:**
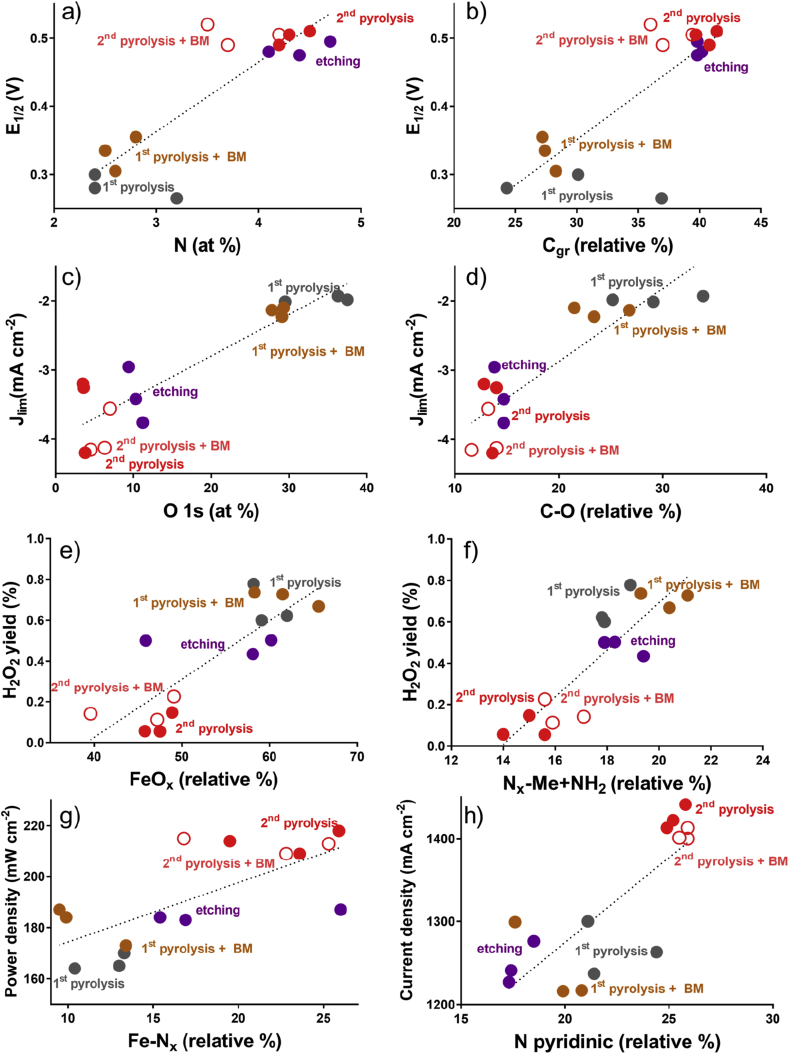
Performance to surface chemistry relationship.

Considering the performances in RRDE and the surface chemistry of the catalysts, it can be noticed that, the increase in total nitrogen that is mainly introduced by etching away surface oxides results in much higher half-wave potential (Fig. 5a). Simultaneously, graphitic content that increases during etching as well has the same positive effect (Fig. 5b). Higher current densities (more negative values) result from a gradual decrease of oxygen that is observed at each step (Fig. 5c), from the first ball milling, through etching which has the largest effect and then by the second pyrolysis. Etching serves as a cleaning step in which amorphous carbon with surface oxides are removed contributing to higher conductivity and higher density of active sites. A similar trend is also observed when limiting current densities are plotted versus C-O peak obtained from C 1s spectrum (Fig. 5d), confirming that surface carbon oxides are the unwanted components of the electrocatalysts chemistry. Finally, a decrease in yield of hydrogen peroxide that is observed with each synthetic step is linked to a decline in the amount of iron oxides (Fig. 5e) and amines (Fig. 5f), which contribute to the same peak as nitrogen coordinated to metal in N 1s spectrum.

Concerning the performance in MFCs, major positive contribution discovered are from iron coordinated to nitrogen extracted from Fe 2p spectra and pyridinic N. Each step of synthesis increases the amount of iron coordinated to nitrogen (Fig. 5g), while etching decreases the amount of pyridinic nitrogen without affecting performance, and major change happens in the second pyrolysis causing much higher current densities registered (Fig. 5h).

## 4 Conclusions

Due to severe limitations, the development of cathode catalysts for microbial fuel systems is of prime importance. As mentioned before, pyrolysis is the most used method and the cheapest in terms of cost of raw materials. Therefore, understanding and quantifying advantages and disadvantages of each step used in synthesis introduce to the electrocatalytic activity is of fundamental importance. Moreover, no universal synthesis to prepare PGM-free catalysts is implemented world-wide, the reproducibility in catalyst synthesis and resulting electrocatalytic activity is critical. The purpose of this work was not only to show the electrocatalytic activity of the Fe-based catalyst in MFCs but to build a direct relationship between electrochemistry and surface chemistry. In this work, an iron catalyst with Nicarbazin precursor was synthesized using Sacrificial Support Method. The synthesis involved 6 steps.

The results showed that during each step in which temperature is involved and utilization of acid during the template removal, the surface chemistry changes significantly as expected. Moreover, the electrochemistry was significantly affected. Both etching and second pyrolysis treatment are increasing performances both in RRDE and in MFC.

Analysis of relationships between surface chemistry and electrochemical performances elucidates several positive and inverse relations. Considering RRDE data analysis, total nitrogen and graphitic carbon contribute to enhance the half-wave potentials during LSVs. Lower content of total oxygen and C-O seems to increase the limiting current of the catalyst. At last, low unwanted hydrogen peroxide produced seems to be related to the low percentage of FeO_x_ and N_x_-M + NH_2_. Focusing on the MFCs performances, pyridinic nitrogen (%) and Fe-N_x_ positively affect the current density and the power output of the MFCs.

As a general consideration, Fe-based catalysts follow a 2x2e-transfer mechanism and produce a very small amount of peroxide which is good for ORR. Once incorporated into an air-breathing cathode and tested in MFCs, it can be noticed that the several synthesis steps contribute to enhancement in the catalyst performance. RRDE results can be used to predict the performances of the catalyst once incorporated into air-breathing cathodes and tested in MFCs. The advantage compared to bare AC was 57% (after the first pyrolysis) and up to 106% (after the second pyrolysis). This demonstrated once again that the utilization of low cost and earth abundant transitional metals as a catalyst for MFCs is justified and the power output is doubled. Maximum power output achieved in this work is 214 ± 5 μWcm^−2^ that is actually in the upper end of the existing literature on PGM-free catalysts. Triplicates batches were produced and surface chemistry and electrochemical performances were similar among the three batches underlining significant reproducibility among the catalyst preparation procedure.
