# Supplementary material for: Influence of platinum group metal-free catalyst synthesis on microbial fuel cell performance
Source: J Power Sources. 2018 Jan 31;375:11–20. doi: 10.1016/j.jpowsour.2017.11.039 (PMC5738968; doi:10.1016/j.jpowsour.2017.11.039)
Supplement: Supplementary material [file mmc1.docx]

# **Influence of platinum group metal-free catalyst synthesis on microbial fuel cell performance**

Carlo Santoro^+^, Santiago Rojas-Carbonell^+^, Roxanne Awais, Rohan Gokhale, Mounika Kodali, Alexey Serov, Kateryna Artyushkova, *Plamen Atanassov

The Department of Chemical and Biological Engineering, Center for Micro-Engineered Materials (CMEM), University of New Mexico, Albuquerque, NM 87131, USA.

^+^ the two authors have equally contributed to the manuscript

*Corresponding author: Plamen Atanassov E-mail: plamen@unm.edu

**Table S1.** Elemental composition of all the samples investigated using XPS

|  | **C %** | **N %** | **O %** | **Fe %** |
| --- | --- | --- | --- | --- |
| **1P-1** | 59.2 | 3.2 | 37.5 | 0.21 |
| **1P-2** | 68 | 2.4 | 29.5 | 0.24 |
| **1P-3** | 61.2 | 2.4 | 36.3 | 0.16 |
| **1PB-1** | 69.6 | 2.5 | 27.8 | 0.26 |
| **1PB-2** | 68.3 | 2.6 | 29.1 | 0.2 |
| **1PB-3** | 67.8 | 2.8 | 29.3 | 0.17 |
| **1PBE-1** | 84.7 | 4.1 | 11.2 | 0.09 |
| **1PBE-2** | 85.2 | 4.4 | 10.3 | 0.08 |
| **1PBE-3** | 85.8 | 4.7 | 9.4 | 0.1 |
| **1PBE2P-1** | 91.8 | 4.3 | 3.8 | 0.15 |
| **1PBE2P-2** | 92.1 | 4.2 | 3.6 | 0.17 |
| **1PBE2P-3** | 92 | 4.5 | 3.5 | 0.13 |
| **1PBE2PB-1** | 90 | 3.7 | 6.3 | 0.13 |
| **1PBE2PB-2** | 91.3 | 4.2 | 4.5 | 0.14 |
| **1PBE2PB-3** | 89.5 | 3.5 | 7 | 0.13 |

**Table S2.** Relative distribution of nitrogen of all the samples using XPS

|  | **N imine** | **N pyridinic** | **N_x_-Fe+amines** | **N-H** | **N gr-N^+^** | **NO_x_** |
| --- | --- | --- | --- | --- | --- | --- |
| **1P-1** | 2.6 | 24.4 | 17.8 | 27.7 | 20.4 | 7 |
| **1P-2** | 3.1 | 21.4 | 17.9 | 28.7 | 21.5 | 7.4 |
| **1P-3** | 2.5 | 21.1 | 18.9 | 29.5 | 22.5 | 5.5 |
| **1PB-1** | 1.7 | 17.6 | 20.4 | 30.6 | 24.4 | 5.3 |
| **1PB-2** | 1.7 | 20.8 | 21.1 | 30.2 | 21.3 | 4.9 |
| **1PB-3** | 2.8 | 19.9 | 19.3 | 28.7 | 23.4 | 5.8 |
| **1PBE-1** | 1.2 | 17.3 | 19.4 | 29.3 | 15.1 | 17.6 |
| **1PBE-2** | 1.2 | 17.4 | 18.3 | 29.3 | 15.6 | 18.2 |
| **1PBE-3** | 0 | 18.5 | 17.9 | 29.2 | 16.4 | 17.9 |
| **1PBE2P-1** | 3.4 | 25.8 | 15 | 29.8 | 18.6 | 7.4 |
| **1PBE2P-2** | 2.3 | 24.9 | 15.6 | 28.8 | 21.5 | 6.9 |
| **1PBE2P-3** | 2.4 | 25.2 | 14 | 30.8 | 19.6 | 8 |
| **1PBE2PB-1** | 2.5 | 25.9 | 17.1 | 28.8 | 19.4 | 6.2 |
| **1PBE2PB-2** | 2.5 | 25.9 | 15.9 | 29.2 | 19.6 | 6.9 |
| **1PBE2PB-3** | 2.3 | 25.5 | 15.6 | 31.4 | 17.9 | 7.3 |

**Table S3.** Relative distribution of carbon of all the samples using XPS

|  | **C gr** | **C-C** | **C-N/ C-O** | | **C=O** | **COOH** |
| --- | --- | --- | --- | --- | --- | --- |
| **1P-1** | 36.9 | 11.8 | 25.2 | 7.4 | 6.5 | 7.3 |
| **1P-2** | 30.1 | 16.1 | 29.1 | 7.1 | 6 | 8.6 |
| **1P-3** | 24.3 | 12.4 | 33.9 | 8 | 8.8 | 8.2 |
| **1PB-1** | 27.4 | 17 | 26.8 | 8 | 8 | 8.8 |
| **1PB-2** | 28.3 | 22.5 | 23.4 | 7.4 | 6.2 | 8.9 |
| **1PB-3** | 27.2 | 25.3 | 21.5 | 7.5 | 6.3 | 8.1 |
| **1PBE-1** | 40.2 | 19.3 | 14.7 | 7.8 | 6.2 | 6.5 |
| **1PBE-2** | 39.8 | 19.1 | 14.7 | 7.7 | 6.5 | 6.3 |
| **1PBE-3** | 39.8 | 18.9 | 13.8 | 8.4 | 6.9 | 5.7 |
| **1PBE2P-1** | 39.7 | 20.8 | 13.6 | 7.6 | 5.1 | 6.3 |
| **1PBE2P-2** | 40.8 | 17.4 | 14 | 8.7 | 5.7 | 6.2 |
| **1PBE2P-3** | 41.4 | 17.6 | 12.8 | 9.1 | 5.9 | 6.1 |
| **1PBE2PB-1** | 37 | 23.4 | 14 | 8.8 | 4.4 | 6.9 |
| **1PBE2PB-2** | 39.4 | 22.6 | 11.6 | 8.5 | 5.2 | 6.3 |
| **1PBE2PB-3** | 36 | 25.3 | 13.2 | 8.5 | 4.6 | 7.3 |

**Table S4.** Relative distribution of iron of all the samples using XPS

|  | **Fe-N_x_** | **FeO_x_** |
| --- | --- | --- |
| **1P-1** | 13 | 87 |
| **1P-2** | 10.4 | 89.6 |
| **1P-3** | 13.3 | 86.7 |
| **1PB-1** | 9.5 | 90.5 |
| **1PB-2** | 9.9 | 90.1 |
| **1PB-3** | 13.4 | 86.6 |
| **1PBE-1** | 15.4 | 84.6 |
| **1PBE-2** | 16.9 | 83.1 |
| **1PBE-3** | 26 | 74 |
| **1PBE2P-1** | 19.5 | 80.5 |
| **1PBE2P-2** | 23.6 | 76.4 |
| **1PBE2P-3** | 25.9 | 74.1 |
| **1PBE2PB-1** | 25.3 | 74.7 |
| **1PBE2PB-2** | 22.8 | 77.2 |
| **1PBE2PB-3** | 16.8 | 83.2 |

**Figure S1.** Disk current (a), peroxide yield (b) and number of electrons transferred (c) for the catalysts synthesized for each step.

**Figure S2.** Disk current for the catalysts synthesized for each step. Batch 1 (a), Batch 2 (b) and Batch 3 (c).

**Figure S3.** Peroxide yield for the catalysts synthesized for each step. Batch 1 (a), Batch 2 (b) and Batch 3 (c).

**Figure S4.** Number of electrons transferred for the catalysts synthesized for each step. Batch 1 (a), Batch 2 (b) and Batch 3 (c).

**
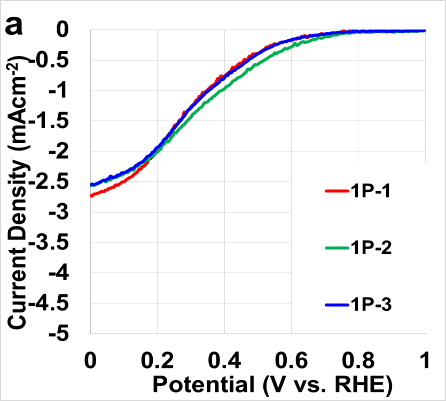

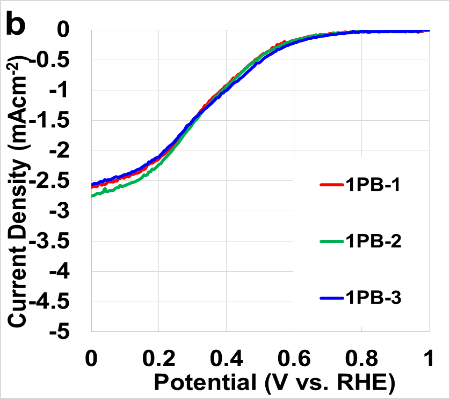

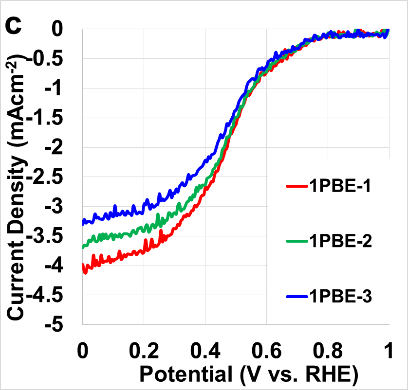

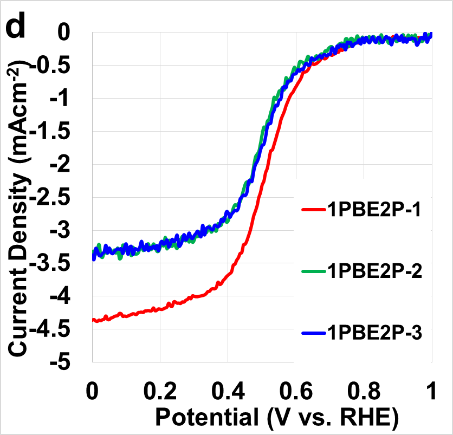

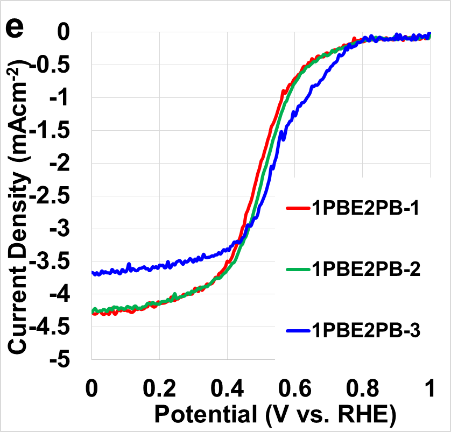
**

**Figure S5.** Disk current for each step 1P (a), 1PB (b), 1PBE (c), 1PBE2P (d) and 1PBE2PE (e) considering the three batches.

**
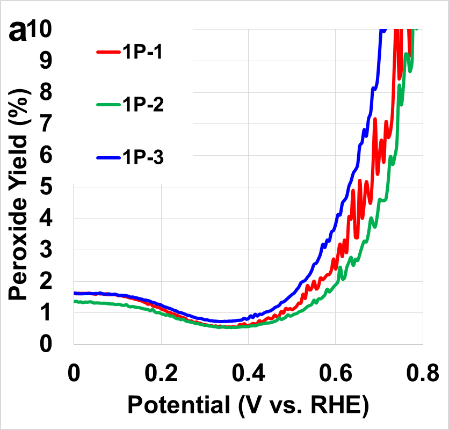

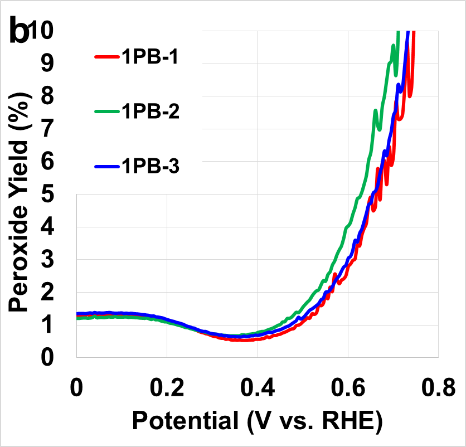

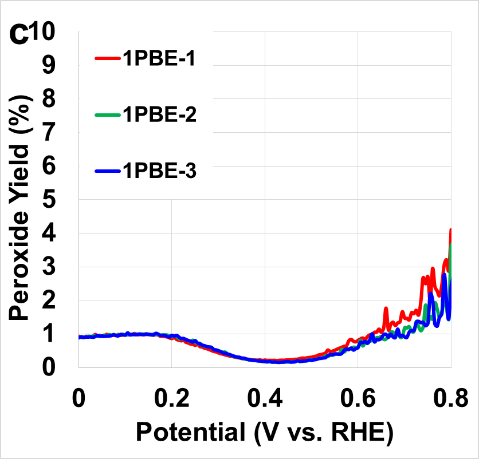

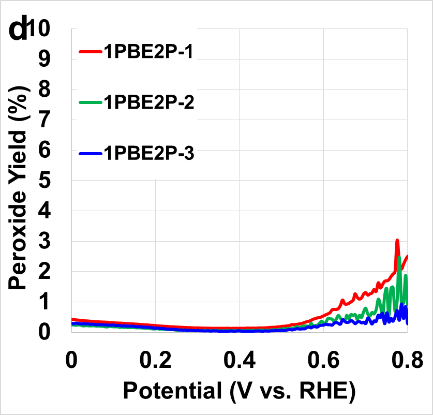

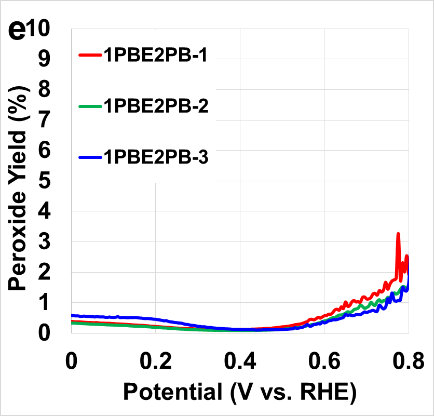
**

**Figure S6.** Peroxide yield for each step 1P (a), 1PB (b), 1PBE (c), 1PBE2P (d) and 1PBE2PE (e) considering the three batches.

**
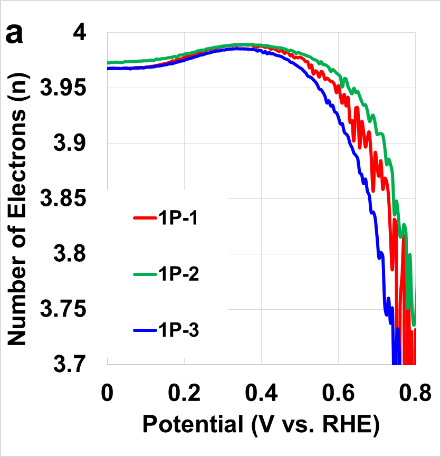

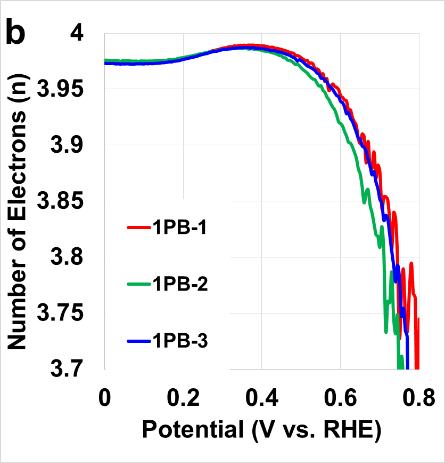

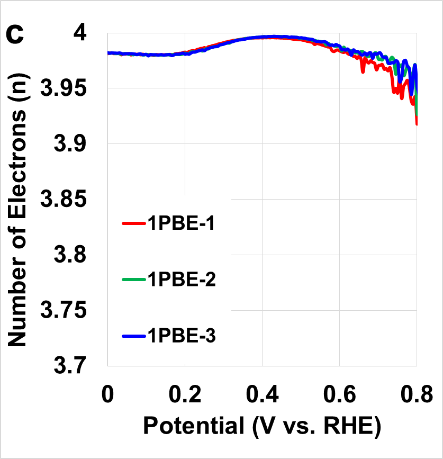
**

**
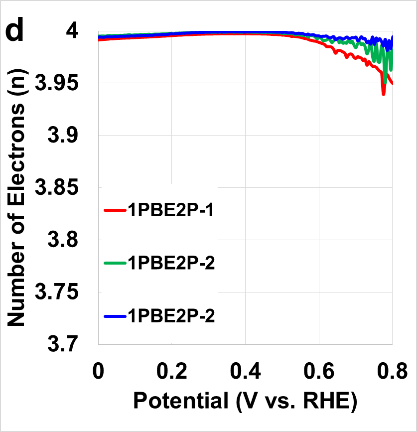

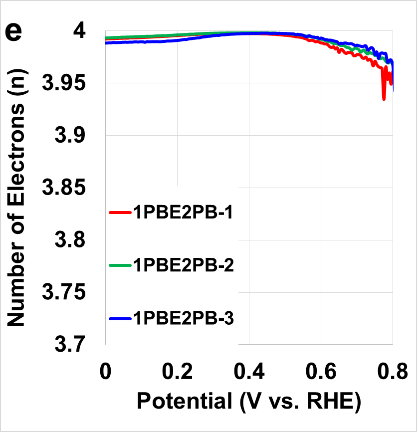
**

**Figure S7.** Number of electrons transferred for each step 1P (a), 1PB (b), 1PBE (c), 1PBE2P (d) and 1PBE2PE (e) considering the three batches.

**Figure S8.** Overall polarization curve (a), power curve (b), anode polarization curve (c) and cathode polarization curve (d) for the MFC with air-breathing cathodes containing catalysts after first pyrolysis (1P).

**Figure S9.** Overall polarization curve (a), power curve (b), anode polarization curve (c) and cathode polarization curve (d) for the MFC with air-breathing cathodes containing catalysts after first pyrolysis and ball milling (1PB).

**Figure S10.** Overall polarization curve (a), power curve (b), anode polarization curve (c) and cathode polarization curve (d) for the MFC with air-breathing cathodes containing catalysts after first pyrolysis, ball milling and etching (1PBE).

**Figure S11.** Overall polarization curve (a), power curve (b), anode polarization curve (c) and cathode polarization curve (d) for the MFC with air-breathing cathodes containing catalysts after first pyrolysis, ball milling, etching and second pyrolysis (1PBE2P).

**Figure S12.** Overall polarization curve (a), power curve (b), anode polarization curve (c) and cathode polarization curve (d) for the MFC with air-breathing cathodes containing catalysts after first pyrolysis, ball milling, etching, second pyrolysis and ball milling (1PBE2PB).
